# Sarcopenia: Loss of mighty armor against frailty and aging

**DOI:** 10.1111/jdi.14067

**Published:** 2023-08-08

**Authors:** Takayoshi Sasako, Kohjiro Ueki

**Affiliations:** ^1^ Department of Diabetes and Metabolic Diseases, Graduate School of Medicine The University of Tokyo Tokyo Japan; ^2^ Diabetes Research Center Research Institute, National Center for Global Health and Medicine Tokyo Japan; ^3^ Department of Molecular Diabetology, Graduate School of Medicine The University of Tokyo Tokyo Japan

## Abstract

The goal of diabetes management is to achieve longevity and quality of life equivalent to those of people without diabetes, and for that, it is now deemed important to pay close attention not only to diabetic vascular complications but also to diabetic comorbidities, as is recommended by the Japan Diabetes Society. In this editorial, we focus on sarcopenia as an important diabetic comorbidity which is an aging‐related phenomenon in skeletal muscle. Taking our recent report on a sarcopenia mouse model and other accumulated evidence into account, we propose the existence of a skeletal muscle‐centered inter‐tissue network that regulates frailty and systemic aging. Sarcopenia is deemed to be a state in which skeletal muscle serving as a protective mighty armor against frailty and systemic aging is lost, and it is vitally important to establish how to recover it and keep it in good shape, so that the goal of diabetes management can be achieved.

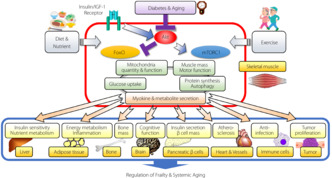

The goal of diabetes management is to achieve longevity and quality of life equivalent to those of people without diabetes, and for that, it is crucial to prevent the development or progression of diabetic vascular complications. Recently, it has increasingly been deemed important to pay close attention also to diabetic comorbidities, such as malignancy, dementia, frailty, and sarcopenia, which are common especially in older patients^1^.

Growing attention has been focused on sarcopenia, meaning in ancient Greek “poverty of flesh”, this being an aging‐related disorder of skeletal muscle characterized by an accelerated loss of muscle mass and function. The diagnosis of sarcopenia requires the measurement of muscle mass, muscle strength, and physical performance, which usually involves an examination of the skeletal muscle mass in the extremities, hand grip strength, and gait speed, respectively. Among the proposed potential mechanisms underlying the development of sarcopenia is the altered action of hormones and growth factors, including insulin and insulin‐like growth factor 1 (IGF‐1)^2^, ^3^. Insulin and IGF‐1 share downstream signaling transducer molecules, while insulin mainly regulates nutrient anabolism, and IGF‐1 mainly regulates cell proliferation. Obesity and aging are known to inhibit insulin action via various mechanisms known as insulin resistance, with IGF‐1 action also assumed to be possibly impaired in tissues with insulin resistance^4^, ^5^.

Frailty is another aging‐related disorder of interest to diabetologists, defined as being in a state vulnerable to stressors, in which even a minor event could result in a deterioration of function without a full recovery to baseline homeostasis being shown^6^. Many organ systems are known to be involved in the development of frailty, with the skeletal muscle being a major contributor^2^, ^6^. Indeed, a diagnosis of frailty requires weight loss, self‐reported exhaustion, low energy expenditure, slow gait speed, and weak grip strength^6^. Weak grip strength and slow gait speed form part of the diagnostic criteria for sarcopenia and frailty. Moreover, while their cut‐off thresholds are not entirely identical among the criteria, it is easy to imagine that loss of muscle mass and volume in sarcopenia leads to weight loss, exhaustion, and low energy expenditure. Thus, it could reasonably be argued that aging in skeletal muscle is closely associated with systemic aging.

We recently reported an animal model of sarcopenia, which had not been established hitherto^5^. By focusing on Akt, a critical downstream kinase of insulin/IGF‐1 signaling^4^, we established skeletal muscle‐specific Akt knockout mice to mimic the insulin resistance in the tissue that is frequently observed in older people. No particular phenotypes are observed when they are young, but they show a reduction in fast‐twitch muscle mass, impaired glucose uptake mediated by insulin, and motor dysfunction as they grow older, proving that these mice serve as a good model of sarcopenia. Interestingly, it is accompanied by osteopenia, a phenomenon of aging in the bone known to co‐exist frequently with sarcopenia^7^. Moreover, their lifespan is shorter than that of the control mice, which means that systemic aging is accelerated, with an increase in deaths from debilitation^5^. Thus, insulin/IGF‐1 signaling in skeletal muscle is deemed a critical target in coping with aging and retaining juvenescence of not only the musculoskeletal system but also the whole body.

Notably, the sarcopenia model mice are susceptible to changes in nutritional status. When fasted for 24 h, they show lower plasma glucose levels, suggesting their inability to maintain glucose levels due to a decreased supply of alanine from skeletal muscle, a major substrate of gluconeogenesis in the liver. In addition, the sarcopenia model mice show an even shorter lifespan under caloric restriction^5^, contrary to the established theory that caloric restriction extends lifespan in lower organisms and even in mammals^8^. Moreover, they show a still shorter lifespan when fed with a high‐fat diet and most of them die from tumors, another of the diabetic comorbidities mentioned above, as do control mice. Indeed, subcutaneously transplanted melanoma cells grow faster in the sarcopenia model mice^5^, consistent with the poor prognosis of cancer in humans with sarcopenic obesity^9^, ^10^.

These results suggest that sarcopenia could be deemed a state of frailty, or a state vulnerable to stressors, including an aging‐related decline in bone mass, under‐nutrition, over‐nutrition, and carcinogenesis. Thus, it appears that skeletal muscle likely serves as a kind of armor against stressors, just like those of gladiators in ancient Rome, which not only works as a nutrient buffer maintaining metabolic homeostasis but also provides resistance to aging and related diseases, possibly via the secretion of some factors or metabolites. It would be also fair to say that sarcopenia is a disease in which such multi‐functional armor is lost. It could be that the sarcopenia model mice may be associated with susceptibility to infection, one of the critical features of frailty^6^ or a decline in cognitive function, another diabetic comorbidity related to aging^1^.

Notably, in this context, the key molecule downstream of Akt turns out to be forkhead box O (FoxO) transcription factor, which is localized out of the nucleus and inactivated when it is phosphorylated by Akt and thus is activated in the absence of Akt. Indeed, additional deletion of skeletal muscle FoxO almost totally, and the administration of a FoxO inhibitor at least partially, reverses the phenotypes due to deletion of skeletal muscle Akt^5^. Another established theory in lower organisms is that suppressed insulin/IGF‐1 signaling is protective against aging via inactivation of Akt and activation of FoxO^8^. On the contrary, suppressed insulin/IGF‐1 signaling rather accelerates aging in the skeletal muscle of mammals via inactivation of Akt and activation of FoxO. Thus, as FoxO inhibition is one of the emerging therapeutic strategies against sarcopenia^5^, it remains to be seen whether FoxO inhibition could also reverse the frailty phenotypes.

Taking these phenotypes of our sarcopenia model mice and other evidence accumulated in this field into account, it is highly probable that skeletal muscle Akt could variably affect the homeostasis of various tissues via myokine and metabolite secretion, consequently regulating frailty and systemic aging. Thus, it is deemed vitally important to elucidate the precise mechanisms through which this skeletal muscle‐centered inter‐tissue network could be impaired by comorbid diabetes or aging and how it could be properly modulated by diet, nutrition, exercise, and other interventions (Figure 1).

**Figure 1:**
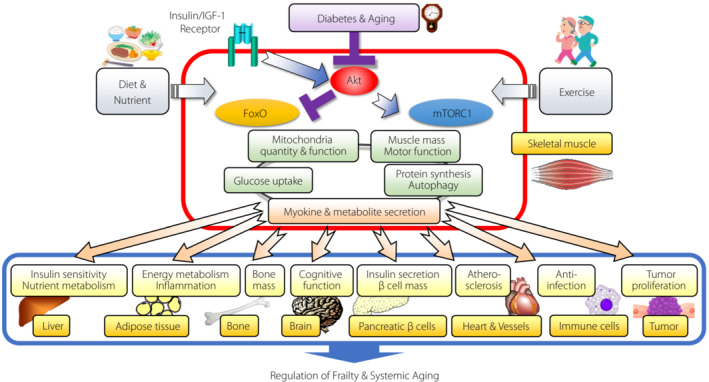
Conceptual scheme of a skeletal muscle Akt‐centered inter‐tissue network mediated by secreted myokines and metabolites to regulate the homeostasis of various tissues and consequently frailty and systemic aging. Skeletal muscle Akt is a critical downstream kinase of insulin/IGF‐1 signaling, and it regulates not only the homeostasis of skeletal muscle itself through various mechanisms but also the homeostasis of other tissues by secreting myokines and metabolites, mainly via suppression of the FoxO pathway and via activation of the mTORC1 pathway as well. However, diabetes and aging suppress the skeletal muscle Akt activity, resulting in the development of frailty and accelerated systemic aging as well as the development of sarcopenia. Diet, nutrition, exercise, and other kinds of therapies are expected to maintain the Akt activity and consequently to keep skeletal muscle functioning as armor against frailty and aging. FoxO, forkhead box O; IGF‐1, insulin‐like growth factor 1; mTORC1, mammalian target of rapamycin complex 1.

Given the decrease in the incidence of diabetic vascular complications and cardiovascular mortality seen in patients with diabetes in past decades, the management of diabetic comorbidities including sarcopenia is becoming increasingly important^11^. Now we need to establish how to recover the armor lost in sarcopenia and how to keep it in good shape, and this will surely help us diabetologists to achieve the goal of diabetes management.

**DISCLOSURE**

The authors declare no conflict of interest. Kohjiro Ueki is an Editorial Board member of *Journal of Diabetes Investigation* and a co‐author of this article. To minimize bias, he was excluded from all editorial decision‐making related to the acceptance of this article for publication.

Approval of the research protocol: N/A.

Informed consent: N/A.

Registry and the registration no. of the study/trial: N/A.

Animal studies: N/A.
